# How immunity from and interaction with seasonal coronaviruses can shape SARS-CoV-2 epidemiology

**DOI:** 10.1073/pnas.2108395118

**Published:** 2021-12-03

**Authors:** Naomi R. Waterlow, Edwin van Leeuwen, Nicholas G. Davies, CMMID COVID-19 Working Group, Stefan Flasche, Rosalind M. Eggo

**Affiliations:** ^a^Centre for Mathematical Modeling of Infectious Disease, London School of Hygiene and Tropical Medicine, London WC14 7HT, United Kingdom;; ^b^Statistics, Modelling and Economics Department, UK Health Security Agency, London NW9 5EQ, United Kingdom

**Keywords:** coronaviruses, immunity, SARS-CoV-2, COVID-19, cross-protection

## Abstract

We hypothesized that cross-protection from seasonal epidemics of human coronaviruses (HCoVs) could have affected severe acute respiratory syndrome coronavirus 2 (SARS-CoV-2) transmission, including generating reduced susceptibility in children. To determine what the prepandemic distribution of immunity to HCoVs was, we fitted a mathematical model to 6 y of seasonal coronavirus surveillance data from England and Wales. We estimated a duration of immunity to seasonal HCoVs of 7.8 y (95% CI 6.3 to 8.1) and show that, while cross-protection between HCoV and SARS-CoV-2 may contribute to the age distribution, it is insufficient to explain the age pattern of SARS-CoV-2 infections in the first wave of the pandemic in England and Wales. Projections from our model illustrate how different strengths of cross-protection between circulating coronaviruses could determine the frequency and magnitude of SARS-CoV-2 epidemics over the coming decade, as well as the potential impact of cross-protection on future seasonal coronavirus transmission.

## Significance

Cross-protection from seasonal epidemics of human coronaviruses (HCoVs) has been hypothesized to contribute to the relative sparing of children during the early phase of the pandemic. Testing this relies on understanding the prepandemic age distribution of recent HCoV infections, but little is known about their dynamics. Using England and Wales as a case study, we use a transmission model to estimate the duration of immunity to seasonal coronaviruses, and show how cross-protection could have affected the age distribution of susceptibility during the first wave, and could alter SARS-CoV-2 transmission patterns over the coming decade.

Due to the relatively short time since severe acute respiratory syndrome coronavirus 2 (SARS-CoV-2) emerged, little is yet known about the duration of infection-induced immunity. While instances of confirmed reinfection of SARS-CoV-2 have been identified (1), these are rare (2), indicating protection lasts for at least 6 mo to 8 mo, which concurs with estimates from prospective studies (3, 4). Cross-protection from seasonal human coronaviruses (HCoVs) could have impacted the transmission dynamics of SARS-CoV-2, and explain the relatively low SARS-CoV-2 infection rate in children (5–8). Since children likely have a higher annual attack rate of endemic HCoVs due to their higher contact rates (9), they may be less susceptible to SARS-CoV-2 due to cross-protection.

In order to evaluate the impacts of cross-immunity, we first need to quantify the immune protection from seasonal coronaviruses. Four coronavirus strains from two different genera are endemic in humans: Two are alphacoronaviruses (HCoV-229E and HCoV-NL63), and two are betacoronaviruses (HCoV-HKU1 and HCoV-OC43); SARS-CoV-2 is a member of the latter genera, as are SARS-CoV-1 and Middle East respiratory syndrome coronavirus (MERS-CoV). In the United Kingdom, seasonal human coronavirus (HCoV) case incidence peaks January–February each year. The first infection with seasonal HCoVs typically occurs in childhood (10), and reinfection with the same strain has been observed within a year (11, 12). However, there are also indications that immunity lasts longer, with few reinfections in a 3-y cohort study (13) and sterilizing immunity to homologous strains of HCoV-229E after 1 y in a challenge study (14).

There may also be cross-protective immunity between seasonal HCoVs and SARS-family coronaviruses following infection. Human sera collected before the SARS-CoV-2 pandemic showed high IgG reactivity to seasonal HCoVs, but also low reactivity to SARS-CoV-2 (15), and SARS-CoV-1 infection induced antibody production against seasonal HCoVs (16, 17). Cross-reactive T cells to SARS-CoV-2 have been found in 20% (18) to 50% (38) of unexposed individuals, with evidence that these responses stem from seasonal coronavirus infection (20). It has also been noted that these are more prevalent in children and adolescents (21).

Cross-protection from seasonal HCoVs may have, therefore, partially shaped the observed epidemiology of SARS-CoV-2. Using England and Wales as a case study, we use dynamic models to estimate 1) the duration of infection-induced immunity to seasonal HCoVs, 2) the ability of potential cross-protection from seasonal HCoVs to explain the age patterns in the first wave of the SARS-CoV-2 pandemic, and 3) the implications of the duration of immunity and potential cross-protection on future dynamics of SARS-CoV-2.

## Results

### Seasonal HCoV and SARS-CoV-2 Epidemic Data.

We extracted monthly, age group–stratified numbers of HCoV positive tests in England and Wales from June 9, 2014 to February 17, 2020 (22), and daily number of COVID-19 deaths in England and Wales during the first wave of the pandemic (March 2, 2020 to June 1, 2020) (23) (*SI Appendix*). The timeframe for the HCoV data is from the first available date until February 2020, to avoid interference from SARS-CoV-2 transmission and reporting.

We fitted a dynamic transmission model using England and Wales as a case study (*SI Appendix*, Fig. S2) using only the seasonal coronavirus model. Following infection, individuals are protected against infection with any seasonal HCoVs, with reinfection possible after a period of temporary but complete immunity. This period is determined by an artificial parameter governing the time to reinfection, due to decaying protection against homotypic viruses, and/or longer-lasting immunity against homotypic viruses but evolutionary change leading to immune escape (24). We do not track individual seasonal HCoV strains, as available data are not subtyped. We therefore assume that individual seasonal HCoV strains have the same parameter values, including *R_0,HCoV_*. Transmission is seasonally forced using a cosine function.

### Seasonal HCoVs Have an *R_0_* of 5.9.

**Fig. 1.**
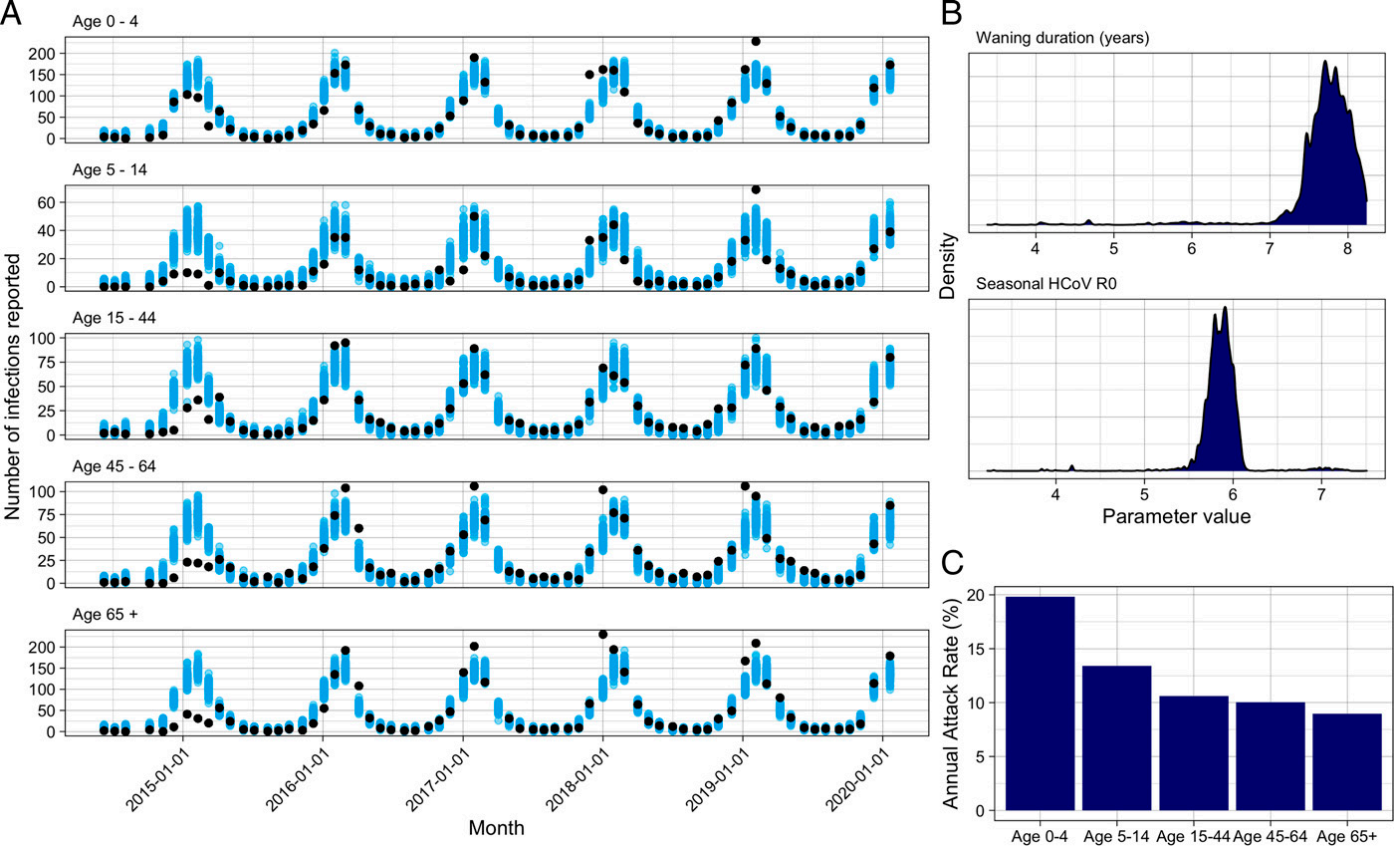
Seasonal HCoV fit. (*A*) Model fit for seasonal HCoV by age. Black dots show reported HCoV cases; blue are 100 random samples from the posterior. (*B*) Posterior distributions for the duration of waning and the *R*_0_ of seasonal HCoV. (*C*) Mean annual attack rate for each age group from 100 samples of the posterior and the last 5 y of the fit.

We fitted the model to the age group–specific seasonal HCoV data from June 9, 2014 until February 17, 2020, and estimated key seasonal HCoV parameters using parallel tempering (25) (Fig. 1). We fitted the artificial immunity parameter, the transmissibility, age-specific reporting proportions, and two seasonal forcing parameters (*SI Appendix*, Table S1). We estimated that the average duration between infection and return to susceptibility for seasonal HCoVs was 7.8 y (95% CI: 6.3 to 8.2) and that the basic reproduction number was 5.9 (95% CI 5.5 to 6.2) (Fig. 1*B*). As a sensitivity analysis, we excluded the first year of surveillance (up until July 2015), due to its different pattern, and here we estimated that the average duration between infection and return to susceptibility for seasonal HCoVs was 4.4 y (95% CI 4.3 to 4.7) and that the basic reproduction number was 3.7 (95% CI 3.6 to 3.8) Further details are given in *SI Appendix*.

### Cross-Protection from Seasonal HCoVs Is Not Sufficient to Explain Age-Specific Patterns of SARS-CoV-2 Infection.

We included SARS-CoV-2 in the model, where each compartment has the state for the combined seasonal HCoVs as well as the state for SARS-CoV-2 (*SI Appendix*, Fig. S2, full model). We included cross-protection that decreases susceptibility to infection by SARS-CoV-2 by an amount, σ, for individuals in the *I_HCoV,i_* or *R_HCoV,i_* states (σ = 0 is no cross-protection, and σ = 1 is full cross-protection). We assume any interaction in the opposite direction would be negligible, due to the low proportion of the population that was infected in the first SARS-CoV-2 epidemic wave.

Using the posterior estimates of the seasonal HCoV parameters and the simulated output as initial states, we continued a simulation of epidemic seasonal HCoVs from January 1, 2020 until June 1, 2020, including the introduction of SARS-CoV-2. Cross-protection from seasonal HCoVs and different mixing patterns (matching observed lockdown patterns, including school closures; see *Materials and Methods*) were the only mechanisms we included that affected infection by SARS-CoV-2, so that we could evaluate the impact of cross-protection on the observed age distribution of cases.

**Fig. 2.**
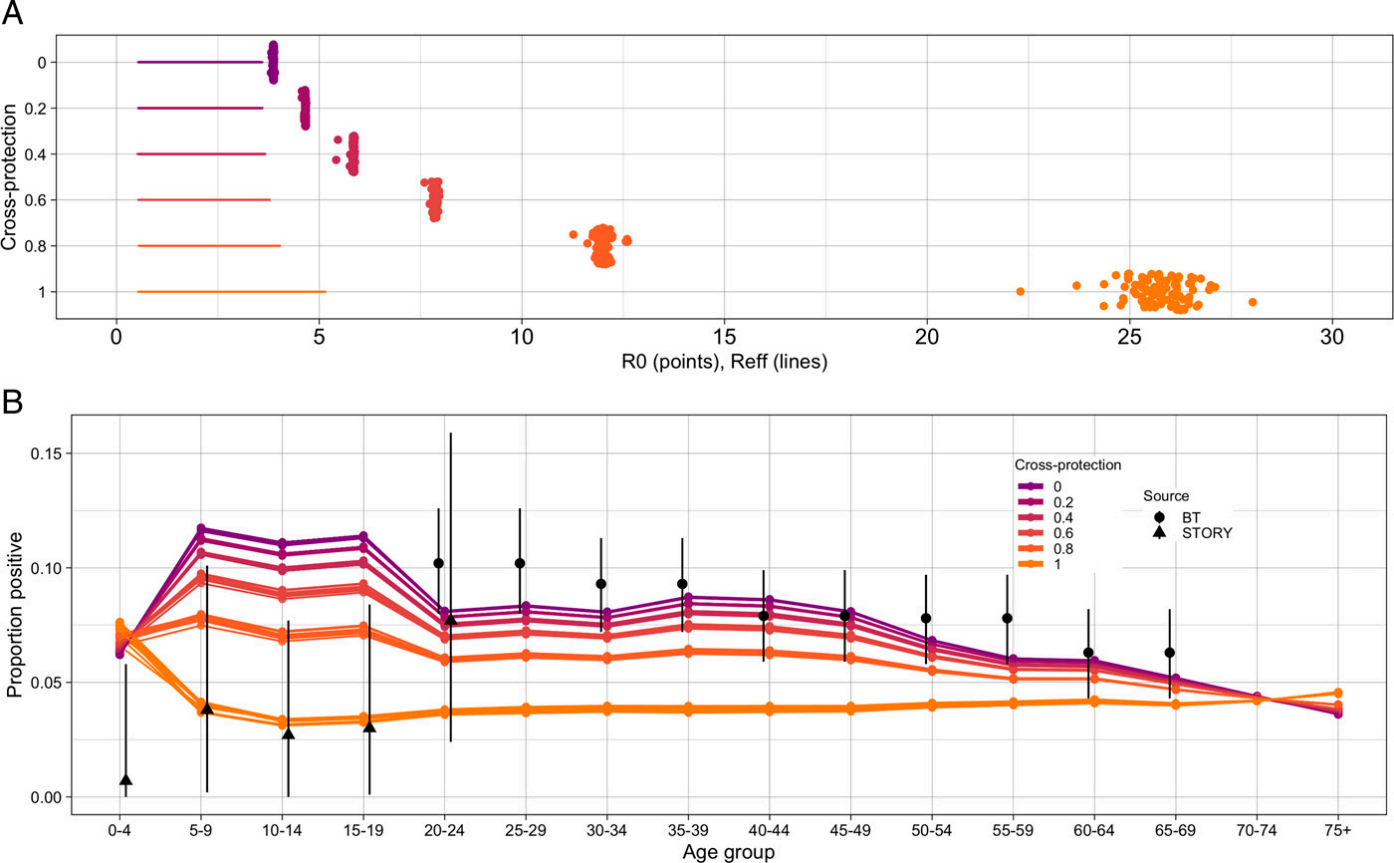
SARS-CoV-2 simulations. (*A*) Estimated *R_0_* values for SARS-CoV-2 with different strengths of cross-protection. Points display the *R_0,C19_*, and lines show the range of *R_eff,C19_* during the simulation. (*B*) Simulated age-specific serology rates for SARS-CoV-2 by the end of May 2020. Sources are Blood and Transplant donors (BT) (26) and the “What’s the STORY” study (STORY) (27).

For values of the cross-protection parameter between σ = 0 and σ = 1, we estimated *R_0,C19_* and the number of introductions of SARS-CoV-2 by fitting the extended model to daily reported COVID-19 deaths. We captured the national lockdown by decreasing contact rates following trends in Google mobility data (28). Our model fits were able to closely match the reported mortality incidence for each value of the cross-protection parameter (*SI Appendix,* Fig. S7). However, the resulting *R_0,C19_* varied widely, reaching over 25 for the strongest cross-protection (Fig. 2A). The corresponding *R_eff,C19_* before the intervention on March 23 ranged between 2.25 and 3.75 (*SI Appendix*).

We then evaluated the age distribution of infections that would be detected by serology by the end of May in our model, across the range of values of the cross-protection parameter (Fig. 2). In simulations with no or low cross-protection, the model predicted larger proportions of children to have been infected than in older age groups, differing from observed data (27, 29). As the strength of interaction increased, the age distribution flattened, and a smaller proportion of children became infected. With complete protection, there was a higher rate in the youngest age groups, which has not been observed (7, 8, 27, 30).

### Future SARS-CoV2 Epidemiology Could Be Shaped by Coronavirus Interactions.

**Fig. 3.**
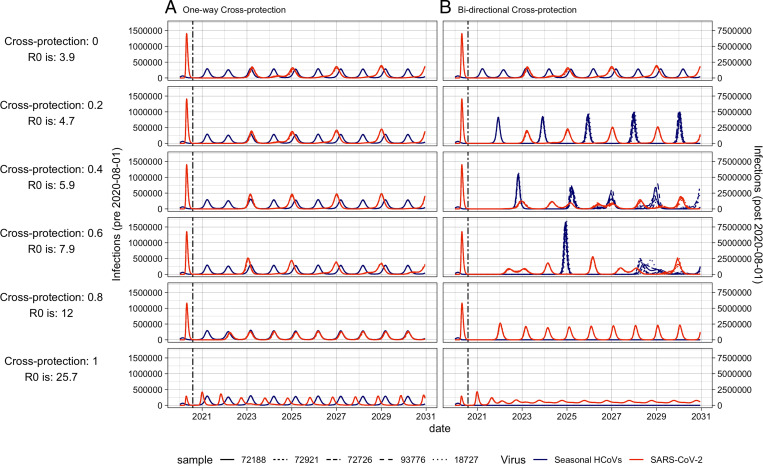
The 10-y forward projections of seasonal HCoV and SARS-CoV-2 epidemics. Red indicates SARS-CoV-2; blue indicates seasonal HCoVs. The dash-dotted vertical line indicates a change in axis scale due to the much larger SARS-CoV-2 pandemic wave, with that to the left of the dashed line marked by the left axis and that to the right marked by the right axis. Cross-protection strength and estimated SARS-CoV-2 *R_0_* for the scenario are shown to the left of the figure. (*A*) Cross-protection from seasonal HCoV to SARS-CoV-2. (*B*) Bidirectional cross-protection. No control measures were included. Different line types show different samples from the posterior of the seasonal HCoV fit.

To determine possible long-term dynamics of interacting coronaviruses, we ran 30-y projections of our model including both HCoVs and SARS-CoV-2, with different assumptions on the strength of cross-protection and whether it acted from HCoV to SARS-CoV-2, or in both directions (Fig. 3). In all scenarios, we assumed no interventions, and used parameters estimated previously. For single-direction cross-protection, annual SARS-CoV-2 epidemics were projected to occur in scenarios with stronger cross-protection, whereas weaker/no cross-protection projected less frequent epidemics. However, strong cross-protection scenarios relied on very high and potentially unrealistic *R_0_*. In weaker cross-protection scenarios, interepidemic periods lasted multiple years following a pandemic. In scenarios with bidirectional cross-protection, SARS-CoV-2 infections also projected frequent epidemics, but led to the seasonal HCoV being disrupted. With low levels of cross-protection, SARS-CoV-2 and seasonal HCoV epidemics alternated, but, as the cross-protection increased, SARS-CoV-2 epidemics became more frequent and outcompeted seasonal HCoV, while a cross-protection of 0.6 resulted in irregular dynamics of the viruses. At higher levels of cross-protection, no seasonal HCoV transmission occurred.

## Discussion

While it was possible to match the COVID-19 mortality data with the full range of cross-protection strengths between seasonal HCoV and SARS-CoV-2, the estimated *R_0,C19_*s were outside of a realistic range for very high values of cross-protection. For example, a recent multisetting study estimated the *R_0,C19_* to be between 3.6 and 7.3 (31). Cross-protection from seasonal HCoVs to SARS-CoV-2 did not fully explain the apparent reduced susceptibility of children to SARS-CoV-2 observed during the first wave in the United Kingdom (6, 8, 27, 29, 30). We estimated that the *R_0_* is 5.7 (95% CI: 5.4 to 6.0) and that time between infection and return to susceptibility was 7.3 y (95% CI: 6.8 to 7.9). We found 12.8% (95% CI: 11.9 to 13.7%) reinfection within 1 y, and the median reinfection time was 5.1 y (95% CI: 4.7 to 5.5 y). Future projections varied in the frequency of SARS-CoV-2 epidemics, with SARS-CoV-2 epidemics every 2 y at low levels of cross-protection, changing to annual epidemics with increased cross-protection. In scenarios with bidirectional cross-protection, epidemics were less predictable, and SARS-CoV-2 outcompeted seasonal HCoVs. Further elucidating possible cross-protection and potential duration of protection is therefore critical for medium-to-long-term projections of SARS-CoV-2 epidemics.

Our estimates for the duration of homotypic protection following HCoV infection are comparable with other estimates, such as a cohort study where 8/216 (3%) confirmed infected individuals were reinfected over 5 y, and the median reinfection time in a study of 10 individuals (32) varied between 30 and 55 mo, depending on strain. However, estimates vary, with a larger study in Michigan estimating mean strain-specific reinfection to be between 19 and 33 mo (33), 19.9% of first infections being reinfected within 6 mo in Kenya (34), and a historical study, of just one seasonal HCoV strain (229E), estimating the time until T cells could no longer neutralize new strains at 8 y to 17 y (24). Other coronaviruses can also give indications on the duration of immunity, with T cells to SARS-CoV-1 detectable up to 11 y postinfection (35). Other modeling studies without age structure have estimated a substantially shorter duration of immunity, at less than a year (36). However, these estimates imply very high annual attack rates, which are not observed in surveillance data, despite coronaviruses often being tested as part of a multiplex respiratory virus PCR panel. Despite differences in the model, such as the focus on seasonal HCoV competition and the exclusion of alphacoronavirus and age structure, our model suggests that a longer period of cross-protection may be more appropriate and should be included in the proposed range of parameters for fitting such models.

Our model suggests that cross-protection between seasonal HCoVs and SARS-CoV-2 could account for some of the reduced susceptibility to infection of children in the first wave of the SARS-CoV-2 epidemic in England and Wales. Specifically, stronger cross-protection decreased the relative susceptibility to infection of children. This is in line with an American study showing that 50% of prepandemic donors had reactive T cells to SARS-CoV-2 (37) and serological markers for a recent seasonal HCoV infection, suggesting that immune responses to seasonal HCoV could elicit cross-protection. Moreover, 48% of uninfected individuals in a cohort from Australia had cross-reactive T cells to SARS-CoV-2, which was strongly correlated with memory T cells against seasonal coronavirus strains (20). Other studies among healthy individuals without SARS-CoV-2 exposure found cross-reactive T cells targeting SARS-CoV-2 in 50% (38), 35% (19), 24% (39), and 20% (18) of participants, suggesting a moderate amount of cross-immunity that likely stems from seasonal coronaviruses. There are indications that these cross-reactive T cells are present at higher frequency in younger vs. older adults (40, 41), correlating with our hypothesis that this could be due to increased infection from seasonal HCoVs. These cross-reactive T cells target the conserved spike protein antigens (40). Antibodies have also been shown to be cross-reactive (42), and back-boosting of anitbodies against conserved HKU1 and OC43 spike antibodies has been observed in COVD-19 infection, with evidence for immunological imprinting (43). The persistence of antibody in the body is more varied and often shorter in duration than T cells (44). Cross-reactive responses have also been identified in other pandemic coronaviruses (15–17, 45), with some also showing cross-protection: SARS-CoV-1 and MERS-CoV T cell epitopes were protective in mice against other human and bat coronaviruses (46), and a lack of HCoV-OC43 antibodies can increase SARS-CoV-2 severity in humans (adjusted odds ratio of 2.68) (47). Cross-neutralizing antibodies across the clade have also been identified (48). However, a longitudinal study showed that, while cross-reactive HCoV antibodies are boosted following SARS-CoV-2 infection, this does not correlate with protection against infection or hospitalization (49), and a lack of antibody-mediated neutralizing cross-protection has been noted between sera from SARS-CoV-1 patients and SARS-CoV-2 (50). In addition, it has been postulated that the small variety in circulating human coronaviruses may have resulted due to competition between coronaviruses filtering out potential emergent coronaviruses (51). Therefore, while there are significant amounts of corroborating evidence that some degree of cross-protection exists, the literature is not conclusive.

Our results indicate that cross-protection from seasonal coronaviruses alone cannot explain reduced susceptibility to infection of children. Other factors are needed to counteract the children’s higher than average exposure probability driven by their contact behavior (9). One mechanism for this could be due to differences in children’s immune systems (52): Children can produce broadly reactive antibodies that have not been influenced by commonly circulating pathogens and have different proportions of blood cell types, such as specific subtypes of memory B cells, and larger populations of IgM-producing cells. Genetic analysis also suggests that cross-reactivity to SARS-CoV-2 antigens cannot fully be explained by seasonal coronaviruses, implying that other unknown viruses/factors may induce cross-immunity (53). We also modeled cross-protection as only reducing susceptibility to infection, whereas there could also be a reduction in transmission and/or disease severity (54–57). Boosting of immunity by multiple infections has also been suggested to influence cross-protection (55), where boosting by repeat seasonal HCoV infections was hypothesized to reduce the cross-protection to SARS-CoV-2. We did not include boosting in our model, due to the added complexity.

The strength and implications of cross-protection between HCoVs and SARS-CoV-2 will become increasingly evident over the coming months and years. Our projections show that, depending on the extent of cross-protection, SARS-CoV-2 could eventually cause annual epidemics (strong cross-protection) or epidemics every 2 y (little cross-protection). If bidirectional cross-protection occurs, SARS-CoV-2 also has the ability to substantially disrupt seasonal HCoV transmission. This is based on our fit of the duration of immunity and the seasonal forcing parameters of seasonal HCoVs, which are likely to differ to some extent in the case of SARS-CoV-2. These scenarios are in line with others (36, 58–60), which suggest that ongoing SARS-CoV-2 transmission is likely. Alternatively, the introduction of SARS-CoV-2 could have different impacts on seasonal HCoVs, for instance, outcompeting betacoronaviruses without affecting the circulation of alphacoronaviruses. A similar dynamic occurred following the 2009 influenza pandemic, where the previous H1N1 strains were replaced by the 2009 H1N1 strain, but H3N2 circulation continued (61, 62). Our modeled projections assumed that no interventions were implemented. However, HCoV circulation was disrupted in winter 2020–2021 (63) likely due to social restrictions designed to curb the transmission of SARS-CoV-2. It is important to understand the longer-term dynamics of SARS-CoV-2, in order to minimize deaths and plan vaccination strategies. From an evolutionary perspective, cross-protection may be a strong driver for selection, so, in the long run, a less transmissible type with greater cross-protection against competing viruses may dominate.

We modeled all seasonal HCoVs as one virus, thereby assuming complete cross-protection between them. There is evidence for cross-protection between seasonal HCoVs and especially within the alpha and beta subtypes, such as the presence of cross-reactive antibodies (64) and evidence from modeling studies (36). While, in some geographies such as the United States and Sweden (36, 65), differing patterns by subtype are observed, this is not the case in the United Kingdom (66). Yet cross-protection may not be complete or may be subtype specific (alphacoronaviruses vs. betacoronaviruses), and hence our assumption could lead to an underestimation of the true duration of protection, because the duration between homotypic infections would be longer than between infections of any subtype. We expect the single-subtype assumption used here to have a relatively small impact on the results of the cross-protection in the first wave of SARS-CoV-2, which uses the average cross-immunity profile at the end of the seasonal HCoV epidemic. However, the assumption may have a larger impact on the longer-term dynamics. We also assumed that the strength of immunity to seasonal HCoVs is constant over repeated infections. An alternative mechanism would be that repeat infections strengthen immunity, as is hypothesized for some respiratory infections, such as respiratory syncytial virus (67), which could have led to a different estimate of reinfection time for seasonal HCoVs. This could therefore result in higher immunity in adults and lower immunity in children and thereby reduce the ability of cross-protection to explain the lower susceptibility to SARS-CoV-2 in children, strengthening our conclusions. Seasonal HCoV cases may have a time-varying reporting rate due to the circulation and testing of other viruses that cause respiratory illness, which could increase reporting or testing during the UK winter respiratory virus season and reduce reporting or testing in the off season. This could affect the amplitude of the epidemics and therefore could inflate the estimate of the seasonal forcing amplitude parameter.

The emergence of SARS-CoV-2 has highlighted our lack of knowledge on coronavirus immunity and long-term dynamics. In our study, we estimate that immunity against seasonal HCoVs can last years; however, by necessity, we made strong assumptions about the cross-immunity between seasonal HCoV strains. Further studies exploring cross-protection between strains for seasonal coronaviruses as well as routinely subtyped surveillance data would help inform future models. Nonetheless, based on the available data, our study indicates that seasonal coronavirus immunity may last multiple years, which should be considered in the planning of subsequent studies. We also conclude that cross-protection from seasonal coronaviruses is not enough to explain the age susceptibility pattern of SARS-CoV-2, indicating other mechanisms must be involved. While serological data could be useful to further evaluate the extent of cross-protection, the reduction in social contacts due to government interventions against SARS-CoV-2 complicates their use. Our models rely heavily on social contact matrices, and getting an accurate understanding of social contacts in the last year comes with many challenges, such as multiple changes in public health interventions with uncertain adherence. Our study highlights the importance of understanding the background environment of coronaviruses for insights into SARS-CoV-2 pandemic progression.

## Materials and Methods

We created a dynamic transmission model that includes cross-protection between seasonal HCoVs and SARS-CoV-2, using the United Kingdom as a case study (Fig. 1). Initially, we fit the model without SARS-CoV-2 and estimated key seasonal HCoV parameters. Next, we simulated SARS-CoV-2 introduction with varying strengths of cross-protection, to investigate the effect on age-specific susceptibility. The model was written in R (68), and the code is available at https://github.com/cmmid/coronavirus_immunity.

### Data.

We extracted the monthly, age group–stratified number of HCoV positive tests in England and Wales between June 9, 2014 and February 17, 2020, reported to Public Health England (PHE) from National Health Service (NHS) and (PHE) laboratories (22). The sources of these cases are “respiratory viral detections by any method (culture, direct immunofluorescence, PCR, 4-fold rise in paired sera, single high serology titre, genomic, electron microscopy, other method and method unknown" (22). Numbers are reported in age groups: 0 y to 4 y, 5 y to 14 y, 15 y to 44 y, 45 y to 64 y, and 65+ y. We did not use data beyond February 2020, as we wanted to estimate seasonal HCoV parameters in the absence of SARS-CoV-2. While we do not have subtype information for the PHE data collected in England and Wales, we know, from studies in Scotland, where subtyping is performed, that there is reasonable consistency in circulating subtypes each year (66).

For SARS-CoV-2, we used the daily number of deaths with a confirmed SARS-CoV-2 positive test in the preceding 28 d from March 2, 2020 until May 31, 2020 reported by the Office for National Statistics (23).

To compare SARS-CoV-2, we used serology from two sources. Firstly, we used data from a study in April and May 2020 of children and young people aged up to 24 in England called “What’s the STORY.” These data assess serology using the Abbott assay, testing for IgG to the SARS-CoV-2 nucleocapsid protein, adjusted for sensitivity and specificity. Secondly, for adults, we used data collected through the UK NHS Blood and Transplant services between March and May 2020 which tested ∼1,000 samples per region in England using the Euroimmun assay and adjusted for the accuracy of the assay and weighted by population.

### Cross-Protection Model.

We created a deterministic compartmental transmission model for coronavirus infections and their interactions. The population are either Susceptible (S), Exposed (E), Infectious (I), or Recovered (R) for both seasonal HCoVs and SARS-CoV-2. The subscripts used are “HCoV” for seasonal HCoVs and “C19” for SARS-CoV-2, with no differentiation between HCoV strains, as the data are not subtyped. Following infection, individuals enter the exposed category and become infected at rates $\lambda_{\mathrm{HCoV},i}$ and $\lambda_{C19,i}$, and individuals enter the infectious category at rates ν_HCoV_ and ν_C19._ They then recover and become fully susceptible again at rate ω. The force of infection for each virus is shown in Eqs. **1** and **2**. Each compartment in the model records the state for SARS-CoV-2 and seasonal HCoVs, with one for each combination of states (*SI Appendix*, Fig. S2), and all durations are exponentially distributed. At any point, individuals can be infected by the other virus, although this is less likely to occur in the I and R categories, determined by the cross-protection parameter, σ. This takes into account both short-term cross-protection from the activation of the immune system and longer-term adaptive immunity. Both modeled viruses (HCoVs and SARS-CoV-2) are seasonally forced with a cosine function, which captures changes in seasonal human behavior and climatic factors.

[1] $$\lambda_{\mathrm{HCoV},i,t}^{\;}\;=\overset{j=\;N}{\underset{j=1}{}}((\frac{A_{\mathrm{HCoV}}*\;\beta_{\mathrm{HCoV}}}{R_{0,\mathrm{HCoV}}})*\cos(\frac{2\pi }{365.25}-\;\phi )\;+\beta_{\mathrm{HCoV}})*\alpha_{i,j}*\;I_{\mathrm{HCoV},j}$$

[2] $$\lambda_{C19,i,t}^{\;}\;=\overset{j=\;N}{\underset{j=1}{}}((\frac{A_{C19}*\;\beta_{C19}}{R_{0,C19}})*\cos(\frac{2\pi }{365.25}-\;\phi )\;+\beta_{C19})*\alpha_{i,j}*\;I_{C19,j}),$$

where *λ* is force of infection, *i* and *j* are age groups, *N* is total number of age groups, *A* is seasonal amplitude, *β* is transmissibility, *α* is contact rates, *I* is number infected, and *ϕ* is timing of seasonal forcing. As the seroprevalence for SARS-CoV-2 stayed below 5% during the modeled period, we assumed that the level of cross-protection conferred by SARS-CoV-2 on HCoV is negligible during the first epidemic wave. Cross-protection was the only mechanism we included for differing susceptibility to SARS-CoV-2 infection by age group, so that we could test whether it explained the observed infection pattern.

The modeled population was stratified into 5-y bands to 75+ y, with constant birth rates, matching death rates, and aging in line with the population of England and Wales (*SI Appendix*, Table S1). Age-assortative mixing was modeled proportionately to patterns of conversational and physical contacts in the POLYMOD study, a European Commission project (9, 69).

We ran an HCoV-only model for 15 y to reach equilibrium, and a further 5 y to generate simulations to match the data on seasonal HCoV cases from June 9, 2014 to February 17, 2020.

### Inferring Seasonal HCoV Parameters.

We used reported seasonal HCoV cases from June 9, 2014 until February 17, 2020 to avoid overlap with SARS-CoV-2, where potential cross-protection could have occurred. We defined a binomial likelihood, where modeled infection incidence maps to reported cases via an age-dependent reporting proportion, $p_{i}$. We assume equal reporting rates in age groups 5 y to 15 y and 45 y to 65 y to reduce the dimensions of the model, as initial fitting suggested these were very similar. The likelihood is therefore

[3] $$\log(L)\;～\;\overset{i=N}{\underset{i=1}{\sum }}\overset{X}{\underset{x=1}{\sum }}k_{x,i}log(p_{i})\;+\;(n_{x,i}-k_{x,i})\log(1-p_{i}),$$

where L is the likelihood, i is the age group to a total of *N* age groups, x are the reported monthly time points, $k_{x,i}$ are the reported HCoV cases by age group, $n_{x,i}$ are the model estimated infections per age group, and $p_{i}$ is the age-specific reporting rate.

We fit the model to the data using parallel tempering, adapted from Vousden et al. (25), which is based on Monte Carlo Markov Chain (MCMC) inference. Unlike MCMC, multiple chains at different temperatures are run in parallel, and swaps of parameter positions between chains are proposed. This allows more comprehensive exploration of the parameter space and allows the chains to move out of local maxima. We ran two sets of 16 chains and confirmed their convergence with the Gelman–Rubin statistic (70), which was <1.1. We then combined the sample from both chains, excluding the burn-in, in order to increase sample size, resulting in 93,900 samples. See *SI Appendix* for more details.

The percentage infected within 1 y and the median duration to reinfection were calculated using distribution and quantile functions from the stats R package (68).

We ran two sensitivity analyses. In the first, we excluded all data before August 2015, as the 2014/2015 year looked abnormal, and could have resulted in a different testing rate, as it was the first year of data collection. In the second, we assumed that 54% of the reported data were betacoronaviruses, as per the Nickbakhsh et al. (66) study from Scotland, and therefore fit to 54% of the original data (rounded to the nearest whole number).

### Simulating SARS-CoV-2 with a Range of Strengths of Cross-Protection.

We drew 100 random samples from the joint posterior distribution and simulated daily deaths reported in the first wave of the SARS-CoV-2 epidemic in England and Wales, between March 2, 2020 and May 31, 2020. We explored the full range of possible cross-protection strengths, in each case, fitting the transmission and introduction rates to the death data using maximum likelihood estimation with a Poisson likelihood. We therefore created 100 simulations of HCoV and SARS-CoV-2 circulation for each strength of cross-protection.

Due to the nonpharmaceutical interventions implemented in this period (“lockdown”), we adjust the contact matrices, which are split into three categories: school contacts, household contacts, and all other contacts. From February 21, 2020, when Google mobility data become available, we adjust our “other” contacts in line with Google mobility data. From February 23, 2020, we eliminate school contacts and assume that all remaining contacts are reduced to 33% of their transmission potential, due to social distancing and behavioral changes (“microdistancing”) (71). SARS-CoV-2 importations occur from February 15, 2020 until lockdown. See *SI Appendix* for more details on the implementation of public health interventions.

To look at the proportion infected during the first wave, we assumed that antibodies would take 3 wk to rise to detectable levels after infection and not wane below the detection threshold during the study period (72).

### Projecting Future Dynamics of SARS-CoV-2 and Seasonal HCoVs.

We ran the model for 30 y, from January 1, 2020 without any changes in contacts, in order to project the future dynamics of SARS-CoV-2. As inputs, we used the estimated parameters from the seasonal HCoV fits, as well as the estimated transmission and introduction rates fitted for each of the samples.

## Supplementary Material

Supplementary File

## Data Availability

Previously published data were used for this work (Respiratory infections: laboratory reports 2015–2020 GOV.UK, https://www.gov.uk/government/publications/respiratory-infections-laboratory-reports-2020 (22), Blood and Transplant donor serology from the National COVID-19 surveillance reports (26) and the What's the STORY study (27). The code and summarized data is available at https://github.com/cmmid/coronavirus_immunity.
