# Supplementary material for: How immunity from and interaction with seasonal coronaviruses can shape SARS-CoV-2 epidemiology
Source: Proc Natl Acad Sci U S A. 2021 Dec 3;118(49):e2108395118. doi: 10.1073/pnas.2108395118 (PMC8670441; doi:10.1073/pnas.2108395118)
Supplement: Supplementary File [file pnas.2108395118.sapp.pdf]

PNAS
www.pnas.org

Supplementary Information for

How immunity from and interaction with seasonal coronaviruses can shape SARS-CoV-2 epidemiology

Naomi R Waterlow[1,*], Edwin van Leeuwen[1,2], Nicholas G. Davies[1], CMMID COVID-19 working group[1], Stefan Flasche[1], Rosalind M Eggo[1].

[1] Centre for Mathematical Modeling of Infectious Disease, London School of Hygiene and Tropical Medicine
[2] Statistics, Modelling and Economics Department, Public Health England, London, UK

Email: naomi.waterlow1@lshtm.ac.uk

1. Data 2
2. Model equations 2
3. R_0 calculations 5
4. Parallel Tempering 7
5. Attack Rates 10
6. Simulating lockdown 11
7. SARS-CoV-2 death simulations 12
7. Sensitivity - Duration of immunity 12
8. Sensitivity - excluding 2014 season 13
9. Sensitivity - only beta-coronaviruses 15
10. Comparison with existing estimates 17

1. Data

We excluded one data point (April 03, 2017) as it was a duplicate of January 30, 2017, and due to the trend in the epidemic we assumed that January 30, 2017 was the correct one.

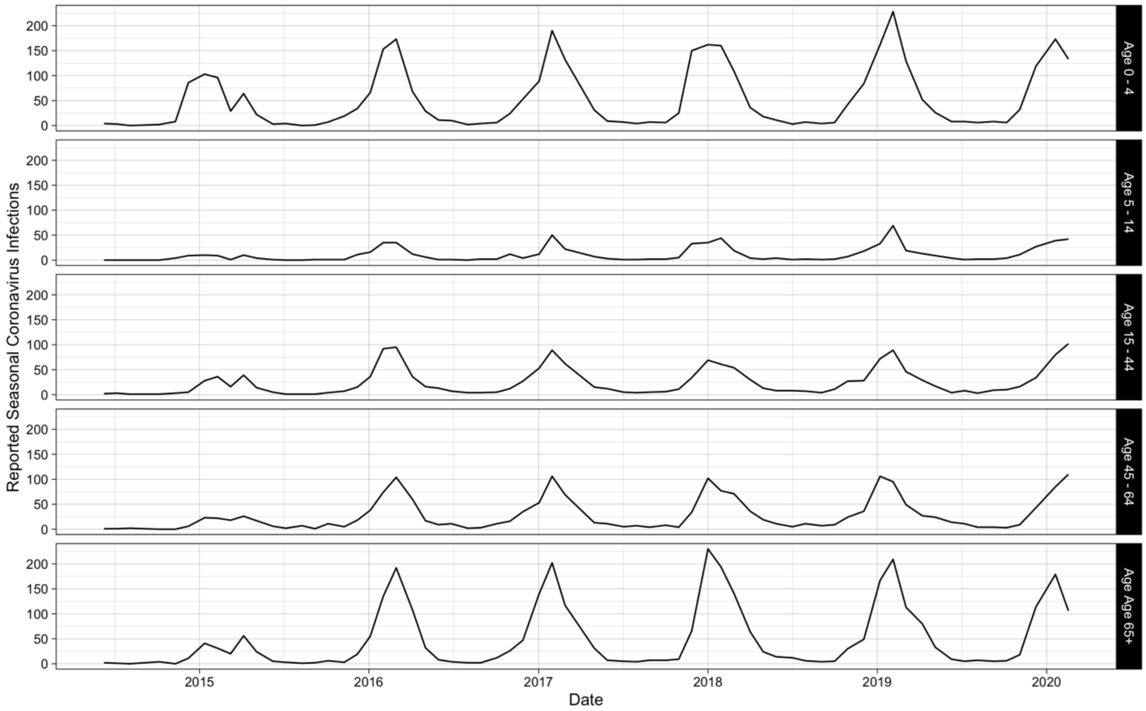

Figure S1: Seasonal Coronavirus reported cases

2. Model equations

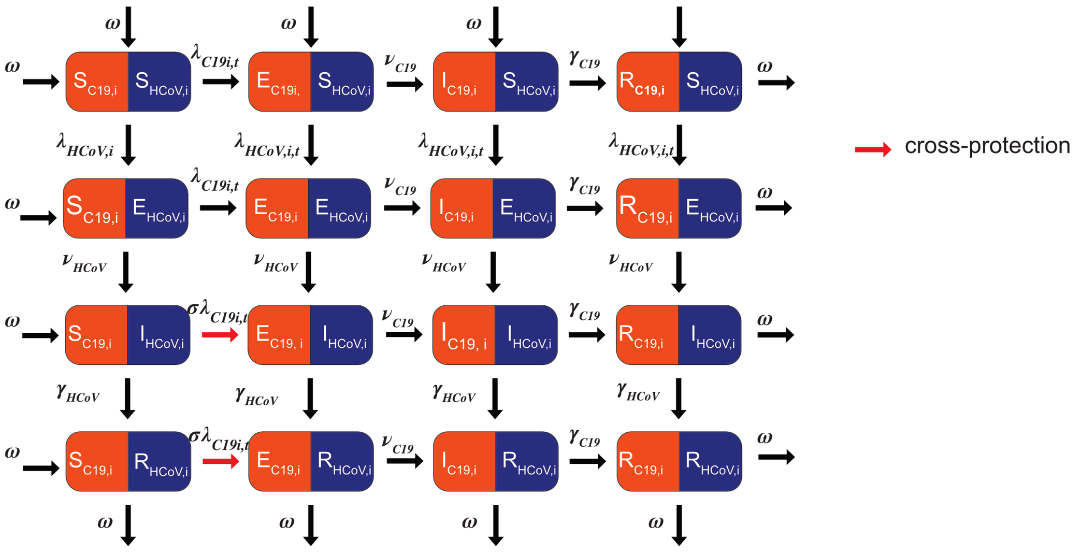

Figure S2: Model Figure

$$\lambda_{HCoV,i,t} = \sum_{j=1}^{j=N}((\frac{A_{HCoV} * \beta_{HCoV}}{R_{0,HCoV}}) * cos(\frac{2\pi}{52*7} - \phi) + \beta_{HCoV}) * \alpha_{i,j} * I_{HCoV,j} \quad (1)$$

$$\lambda_{C19,i,t} = \sum_{j=1}^{j=N}((\frac{A_{C19} * \beta_{C19}}{R_{0,C19}}) * cos(\frac{2\pi}{52*7} - \phi) + \beta_{C19}) * \alpha_{i,j} * I_{C19,j}) \quad (2)$$

$$\frac{dSS_i}{dt} = -\lambda_{C19,i}\, SS_i - \lambda_{HCoV,i,t} SS_i + \omega_{C19} RS_i + \omega_{HCoV} SR_i + \mu_{a,i} SS_{i-1} - \mu_{a,i+1} SS_i + \mu_{b,i} - \mu_{d,i} SS_i$$

$$\frac{dES_i}{dt} = -\nu_{C19} ES_i - \lambda_{HCoV,i,t} ES_i + \lambda_{C19,i}\, SS_i + \omega_{HCoV} ER_i + \mu_{a,i} ES_{i-1} - \mu_{a,i+1} ES_i - \mu_{d,i} ES_i$$

$$\frac{dIS_i}{dt} = -\gamma_{C19} IS_i - \lambda_{HCoV,i,t} IS_i + \nu_{C19} ES_i + \omega_{HCoV} IR_i + \mu_{a,i} IS_{i-1} - \mu_{a,i+1} IS_i - \mu_{d,i} IS_i$$

$$\frac{dRS_i}{dt} = -\omega_{C19} RS_i - \lambda_{HCoV,i,t} RS_i + \gamma_{C19} IS_i + \omega_{HCoV} RR_i + \mu_{a,i} RS_{i-1} - \mu_{a,i+1} RS_i - \mu_{d,i} RS_i$$

$$\frac{dSE_i}{dt} = -\lambda_{C19,i}\, SE_i - \nu_{HCoV} SE_i + \omega_{C19} RE_i + \lambda_{HCoV,i,t} SS_i + \mu_{a,i} SE_{i-1} - \mu_{a,i+1} SE_i - \mu_{d,i} SE_i$$

$$\frac{dEE_i}{dt} = -\nu_{C19} EE_i - \nu_{HCoV} EE_i + \lambda_{C19,i}\, SE_i + \lambda_{HCoV,i,t} ES_i + \mu_{a,i} EE_{i-1} - \mu_{a,i+1} EE_i - \mu_{d,i} EE_i$$

$$\frac{dIE_i}{dt} = -\gamma_{C19} IE_i - \nu_{HCoV} IE_i + \nu_{C19} EE_i + \lambda_{HCoV,i,t} RS_i + \mu_{a,i} IE_{i-1} - \mu_{a,i+1} IE_i - \mu_{d,i} IE_i$$

$$\frac{dRE_i}{dt} = -\omega_{C19} RE_i - \nu_{HCoV} RE_i + \gamma_{C19} IE_i + \lambda_{HCoV,i,t} RS_i + \mu_{a,i} RE_{i-1} - \mu_{a,i+1} RE_i - \mu_{d,i} RS_i$$

$$\frac{dSI_i}{dt} = -\sigma\, \lambda_{C19,i}\, SI_i - \gamma_{HCoV} SI_i + \omega_{C19} RI_i + \nu_{HCoV} SE_i + \mu_{a,i} SI_{i-1} - \mu_{a,i+1} SI_i - \mu_{d,i} SI_i$$

$$\frac{dEI_i}{dt} = -\nu_{C19} EI_i - \gamma_{HCoV} EI_i + \sigma\, \lambda_{C19,i}\, SI_i + \nu_{HCoV} EE_i + \mu_{a,i} EI_{i-1} - \mu_{a,i+1} EI_i - \mu_{d,i} EI_i$$

$$\frac{dII_i}{dt} = -\gamma_{C19} II_i - \gamma_{HCoV} II_i + \nu_{C19} EI_i + \nu_{HCoV} IE_i + \mu_{a,i} II_{i-1} - \mu_{a,i+1} II_i - \mu_{d,i} II_i$$

$$\frac{dRI_i}{dt} = -\omega_{C19} RI_i - \gamma_{HCoV} RI_i + \gamma_{C19} II_i + \nu_{HCoV} RE_i + \mu_{a,i} RI_{i-1} - \mu_{a,i+1} RI_i - \mu_{d,i} RI_i$$

$$\frac{dSR_i}{dt} = -\sigma\, \lambda_{C19,i}\, SR_i - \omega_{HCoV} SR_i + \omega_{C19} RR_i + \gamma_{HCoV} SI_i + \mu_{a,i} SR_{i-1} - \mu_{a,i+1} SR_i - \mu_{d,i} SR_i$$

$$\frac{dER_i}{dt} = -\nu_{C19} ER_i - \omega_{HCoV} ER_i + \sigma\, \lambda_{C19,i}\, SR_i + \gamma_{HCoV} EI_i + \mu_{a,i} ER_{i-1} - \mu_{a,i+1} ER_i - \mu_{d,i} ER_i$$

$$\frac{dIR_i}{dt} = -\gamma_{C19} IR_i - \omega_{HCoV} IR_i + \nu_{HCoV} ER_i + \gamma_{HCoV} II_i + \mu_{a,i} IR_{i-1} - \mu_{a,i+1} IR_i - \mu_{d,i} IR_i$$

$$\frac{dRR_i}{dt} = -\omega_{C19} RR_i - \omega_{HCoV} RR_i + \gamma_{C19} IR_i + \gamma_{HCoV} RI_i + \mu_{a,i} RR_{i-1} - \mu_{a,i+1} RR_i - \mu_{d,i} RR_i$$

States

The first letter of the state indicates the state for SARS-CoV-2, the second letter indicates the state for HCoVs.

S: Susceptible
E: Exposed
I: Infected
R: Recovered

Subscripts
C19: SARS-CoV-2
HCoV: Seasonal HCoVs
i, j: age groups
t: time

Table S1: Model parameters

Parameter type	Parameter	Symbol	Value	Reference
Seasonal HCoV	Basic Reproduction number	$R_{0,HCoV}$	Fitted. Limits: 1-8.5	Wide range
	Transmission rate	β_{HCoV}	Fitted	Based on R_0 calculation (supplement)
	Latent period	$1/\nu_{HCoV}$	2.5 days	1,2
	Duration of infectiousness	$1/\gamma_{HCoV}$	5 days	1
	Incubation period (time to symptoms)	$1/\delta1_{HCoV}$	2 days	3
	Reporting delay (symptom to report)	$1/\delta2_{HCoV}$	3 days	Based on influenza model[4]
	Age-specific reporting proportion	$\mu_{HCoV,i}$	Fitted. Limits 0-1. Proposed on log odds scale.	
	Seasonal forcing amplitude	A	Fitted. Limits: 0 - 2	
	Seasonal forcing timing	ϕ	Fitted. Limits: -(52*7)-(52*7)	
	Immunity duration	$1/\omega$	Fitted. Limits: 100 - 3000	Covers range of 100 days to over 8 years
SARS-CoV-2	Basic Reproduction number	$R_{0,C19}$	Fitted	Based on R_0 calculations (see supplement).
	Transmission rate	β_{C19}	Fitted	
	Effective Reproduction Number	$R_{eff,C19}$	Fitted	R_0 * proportion susceptible (see supplement)
	Latent period	$1/\nu_{C19}$	3 days	1

	Duration of infectiousness	$1/\gamma_{C19}$	5 days	5
	Time between infectiousness (entering I compartment) and death	$1/\delta_{C19}$	22 days (split over two compartments, Erlang distributed)	6
	Age-specific infection fatality proportions (age groups 0-4, 5-14, 15-44, 45-64, 65+)	$\mu_{C19,i}$	0.00004, 0.00004, 0.00024, 0.00441, 0.06720	As in Levin[7], weighted by model population sizes
	Adult (15-64 years) introduction rate	$1/\eta$	Fitted	
	Duration of immunity	$1/\omega$	Fitted	Assumed equal to HCoV waning rate
Demographic	Birth rate	μ_b	640 370 per year	ONS statistical bulletin 2019[8]
	Death rate	μ_d	640 370 per year	Equal to birth rate to maintain constant population
	Population size	N	59 439 840	ONS 2019 population estimates for England and Wales, 5-year age bands[9]

3. R_0 calculations

We used the method described by Diekmann *et al.* (2009)[11] to calculate the R_0 for each virus. The dominant eigenvalue of the matrix is the R_0 of the matrix $-T\Sigma^{-1}$, where $T$is the transmission part of the Jacobian matrix, describing new infections and Σ is the transition part, describing changes in the infectious state. See reference for further details. For seasonal HCoVs we used the base transmission rate.

For each age group, compartments in the matrix are SE and SI, and ES and IS, as we calculate the R_0 assuming no cross-protection. The first row/column represents the E compartments, and the second row represents the I compartment for the first age group. Only the transmissions for the first age group are shown. Three dots (…) represent the pattern continuing, one dot (.) represents equations not shown because they do not refer to the first age group.

$$
\mathrm{T}_{C19} = \left| \begin{array}{cccccc}
0 & \beta_{C19}\,\alpha_{i,j} & 0 & \beta_{C19}\,\alpha_{i,j} & \ldots & \beta_{C19}\,\alpha_{i,j} \\
0 & 0 & 0 & 0 & \ldots & 0 \\
0 & \beta_{C19}\,\alpha_{i,j} & . & . & . & . \\
0 & 0 & . & . & . & . \\
0 & \beta_{C19}\,\alpha_{i,j} & . & . & . & . \\
\ldots & \ldots & . & . & . & . \\
0 & \beta_{C19}\,\alpha_{i,j} & . & . & . & .
\end{array} \right|
$$

$$
\mathrm{T}_{HCoV} = \begin{vmatrix}
0 & \beta_{HCoV}\,\alpha_{i,j} & 0 & \beta_{HCoV}\,\alpha_{i,j} & \dots & \beta_{HCoV}\,\alpha_{i,j} \\
0 & 0 & 0 & 0 & \dots & 0 \\
0 & \beta_{HCoV}\,\alpha_{i,j} & . & . & . & . \\
0 & 0 & . & . & . & . \\
0 & \beta_{HCoV}\,\alpha_{i,j} & . & . & . & . \\
\dots & \dots & . & . & . & . \\
0 & \beta_{HCoV}\,\alpha_{i,j} & . & . & . & .
\end{vmatrix}
$$

$$
\Sigma_{C19} = \begin{vmatrix}
-\nu_{C19} - \mu\alpha_i & 0 & 0 & \dots & 0 \\
-\nu_{C19} & -\gamma_{C19} - \mu\alpha_i & 0 & \dots & 0 \\
\mu\alpha_{i+1} & 0 & . & . & . \\
0 & \mu\alpha_{i+1} & . & . & . \\
0 & 0 & . & . & . \\
\dots & \dots & . & . & . \\
0 & 0 & . & . & .
\end{vmatrix}
$$

$$
\Sigma_{HCoV} = \begin{vmatrix}
-\nu_{HCoV} - \mu\alpha_i & 0 & 0 & \dots & 0 \\
-\nu_{HCoV} & -\gamma_{HCoV} - \mu\alpha_i & 0 & \dots & 0 \\
\mu\alpha_{i+1} & 0 & . & . & . \\
0 & \mu\alpha_{i+1} & . & . & . \\
0 & 0 & . & . & . \\
\dots & \dots & . & . & . \\
0 & 0 & . & . & .
\end{vmatrix}
$$

For the SARS-CoV-2 simulations we calculated the R_{eff} through time (Figure S1), which is influenced by the R_0, the level of cross-protection and the seasonal HCoV circulation. The R_{eff} is the largest eigenvalue of the NGM where each row is multiplied by the proportion susceptible in that age group. Estimates for the $R_{effective}$ in the UK of SARS-CoV-2 before lockdown were between 2.25 and 3.75, so we used these as boundaries. During this period it was only the highest level of cross-protection that did not have an appropriate R_0.

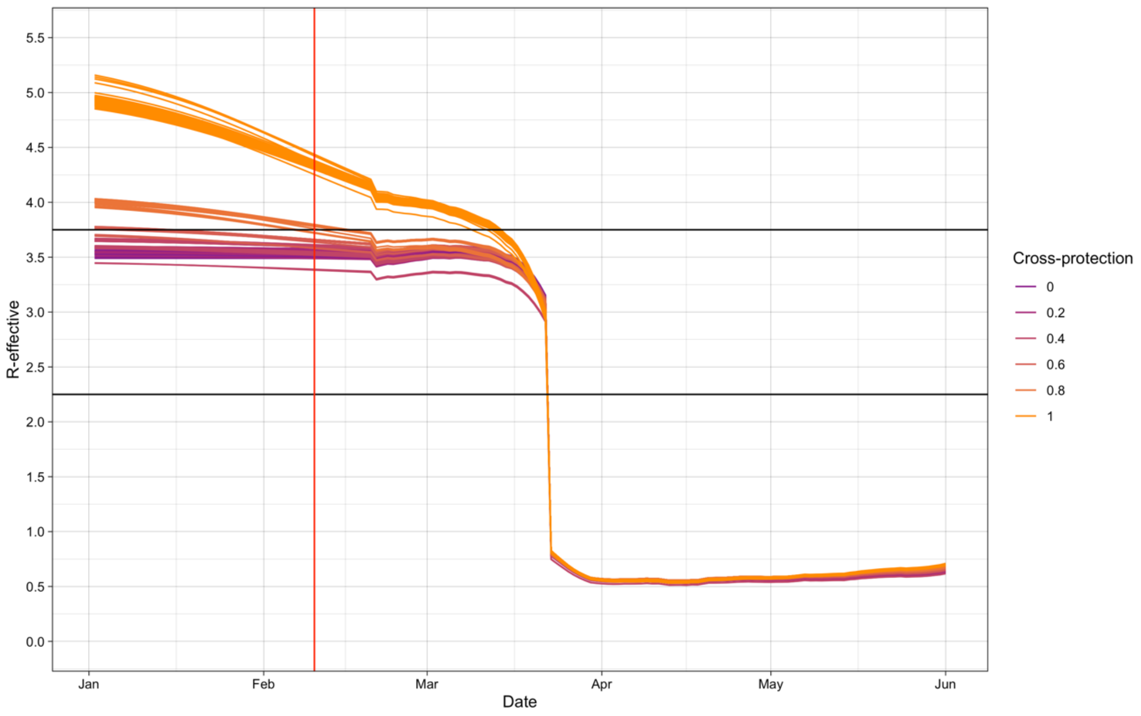

Figure S3: $R_{effective}$ values over time for SARS-CoV-2. Blue lines indicate $R_{effective}$ for simulations at different levels of cross-protection. Black lines show the $R_{effective}$ limits of 2.25 and 3.75 and the red line shows the date of SARS-CoV-2 introduction.

4. Parallel Tempering

We proposed chains to swap with the chain of the next lowest temperature every 5 iterations of the MCMC. The highest and lowest temperatures were fixed at 1000 and 1 (the null chain). The number of chains was then adjusted to achieve an acceptance rate of swaps of between 0.15 and 0.25 . Swaps were accepted based on our swapping equation, adapted from Vousden *et al*[12] following the equation:

$$R = e^{\left(\frac{(LL(i) - LL(j))}{\tau_j - \tau_i}\right)}$$

Where

$$\tau_i = \frac{1}{T_i}$$

And T_i is the temperature of chain i, and LL(i) is the log likelihood of chain i.

We then ran the parallel tempering algorithm from multiple different start values, each with 16 chains. Within each chain parameters were proposed using a covariance matrix. This resulted in two converged chains, which we confirmed by checking that the Gelman-Rubin statistic[13] was <1.1. We discarded 12000 iterations of each as burn in and then combined the samples from the two converged regions to increase the sample size. A set of chains is shown in Figure S2. Figure S3 shows the posterior distribution for all parameters and they are summarised in Table S1.

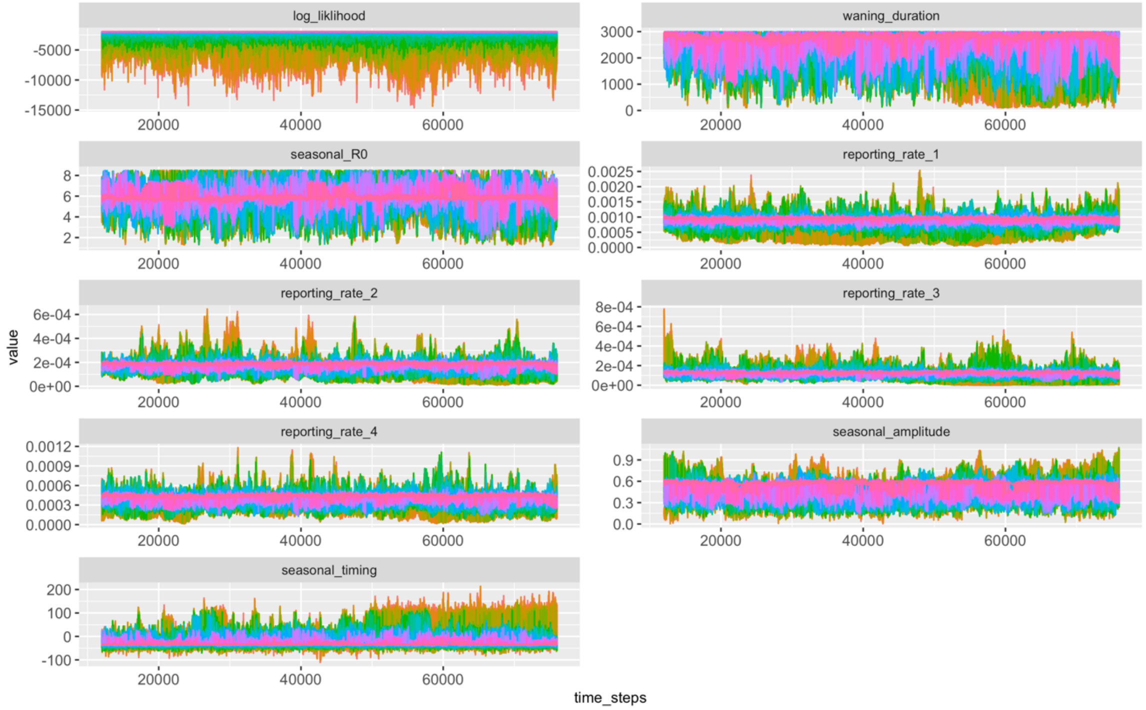

Figure S4: Trace plots of one replicate showing all 16 chains. Each colour is one chain, with bright pink being the coolest (null) chain.

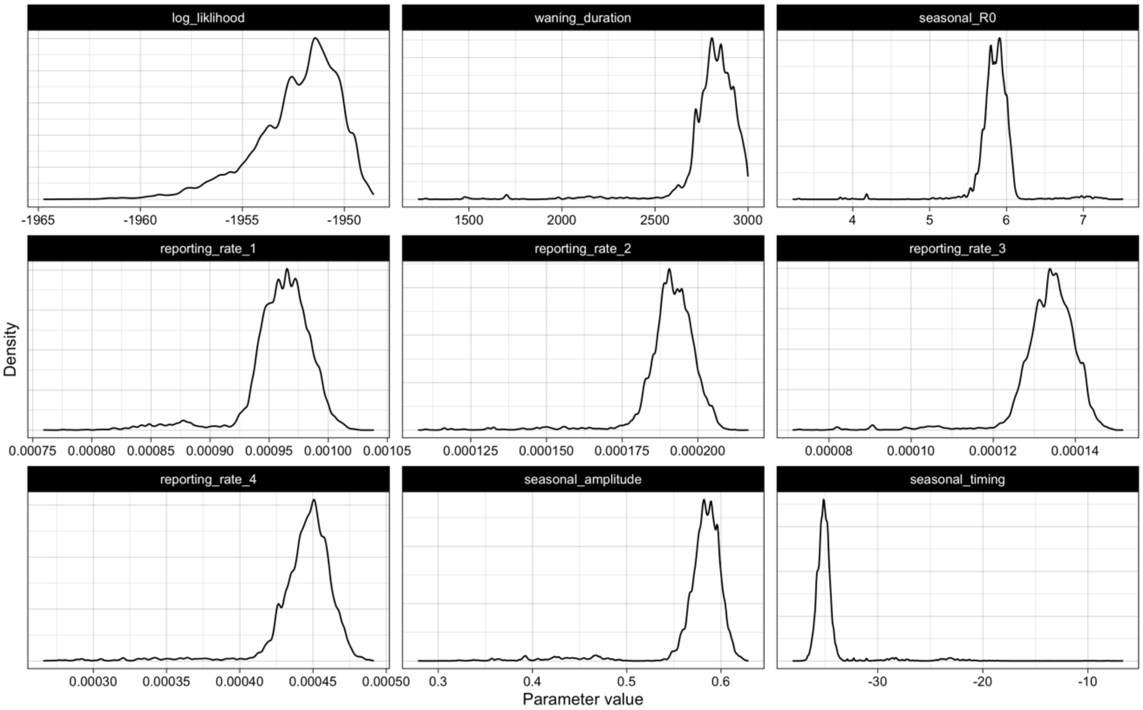

Figure S5: Posterior distributions of fitted parameters for the HCoV fit.

Table S2: Median and 95% quantiles of the posterior distributions of fitted HCoV parameters.

Parameter	Symbol	Median (95% CI)
Basic Reproduction number	$R_{0,HCoV}$	5.9 (5.5 - 6.2)
Immunity duration	ω	7.8 (6.3 - 8.2)
Age-specific reporting proportion 0-4	$\mu_{HCoV,i}$	0.00096 (0.00087 - 0.00100)
Age-specific reporting proportion 5 - 14, 45-64	$\mu_{HCoV,i}$	0.00019 (0.00016 - 0.00020)
Age-specific reporting proportion 15-44	$\mu_{HCoV,i}$	0.00013 (0.00011 - 0.00014)
Age-specific reporting proportion 65+	$\mu_{HCoV,i}$	0.00058 (0.00043 - 0.00061)
Seasonal forcing amplitude	A	0.58 (0.42 - 0.61)
Seasonal forcing timing	ϕ	-35.1 (-36.2 - -24.1)

Figure S6 shows a heatmap of the likelihood of the model at different value of R_0 and durations of immunity waning. One sample from the posterior of the fit was taken, and all parameter values apart from the R_0 and the duration of waning were kept constant. The log likelihood was then calculated for each combination of R_0 and waning durations. The fitted value is shown with a +.

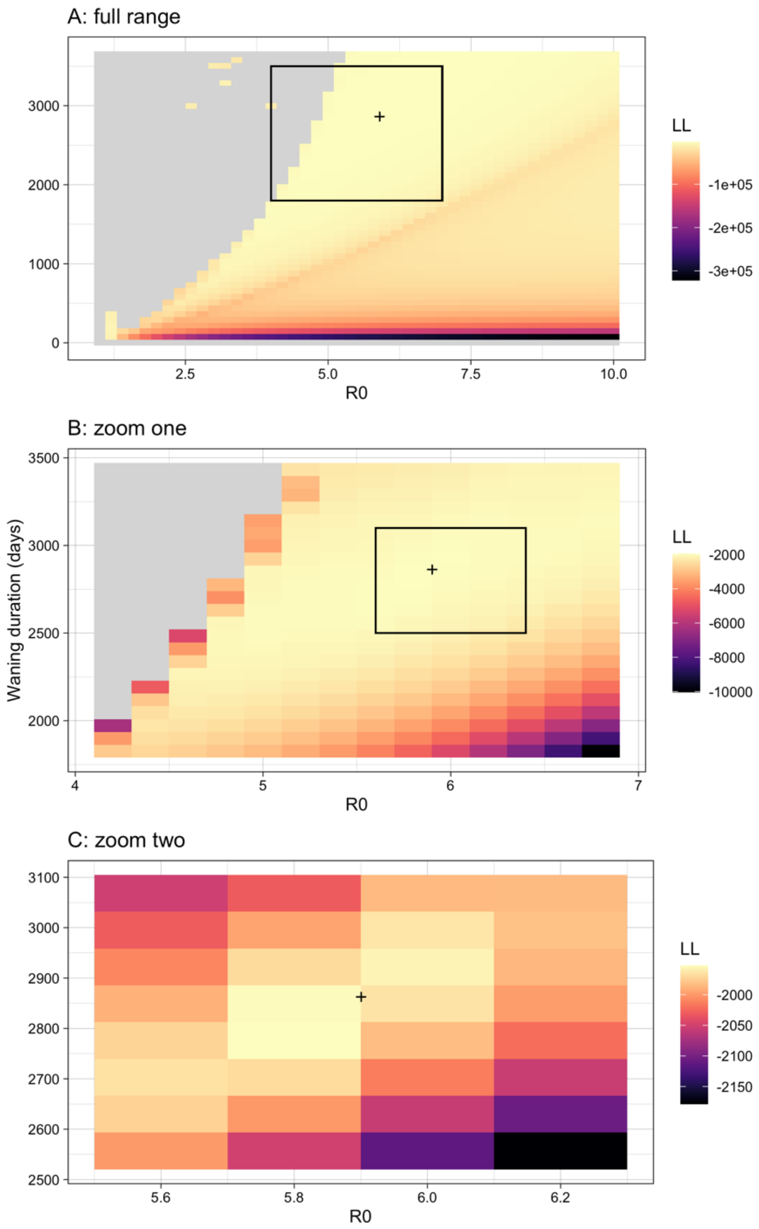

Figure S6: Likelihood plane with varying R_0 and duration of waning. A) shows the full range B) and C) show zoomed in areas. Colour indicates the likelihood value. The black rectangles show the area zoomed in on and the '+' symbol shows the estimated values from the parallel tempering fits.

5. Attack Rates

Average annual attack rates for the seasonal HCoV are shown in Table S2. This is the mean attack rate for each age group, averaged over 100 samples from the joint posterior and the last 5 years in our seasonal HCoV fit.

Table S3: Mean attack rates by age group. Values given are mean across 100 random samples from the posterior and the last 5 years of the seasonal HCoV fit.

Age group	Attack rate (%)
< 5	19.9
5 - 14	13.9
15 - 44	11.0
45 - 64	10.4
65+	9.3

6. Simulating lockdown

Due to the non-pharmaceutical interventions implemented in this period ("lockdown"), we adjust the contact matrices, which are split into three categories: school contacts, household contacts and other contacts. Other contacts included all other categories reported in the polymod dataset. We then adjusted the contacts as follows.

- From February 21, 2020 (when google mobility data first becomes available), we adjust our 'other' contacts group by the average change in retail/recreation, workplace, grocery/pharmacy and transit stations, according to the Google Mobility UK records
- From March 23, 2020 (lockdown including school closures), school contacts are reduced to 0 with no re-attribution of those contacts.
- From March 23, 2020 (lockdown), Other and household contacts are multiplied by a 'social distancing factor' which we set at 0.33. This simulates other interventions such as social distancing, increased hand washing and mask wearing, and we chose the value based on being within a plausible range and appropriate looking simulations. We adapt households as the original contact matrix includes all household interactions, including visitors.
- Importations occur from the date of SARS-CoV-2 introduction (February 15, 2020) in the UK until the March 23, 2020 (lockdown). The date of SARS-CoV-2 introduction was chosen as a plausible value that allowed the simulated deaths to peak at the right time of year.

7. SARS-CoV-2 death simulations

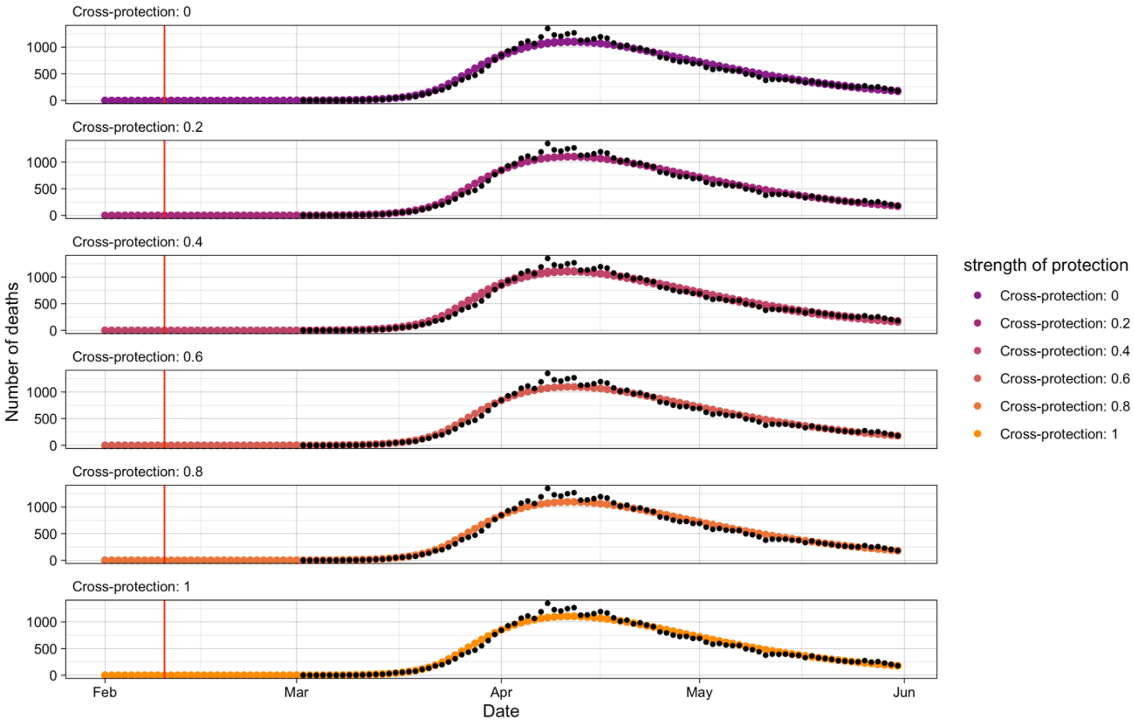

Figure S7. Fit to deaths. SARS-CoV-2 death simulations compared to the death data, with varying strength of cross-protection. The red line indicates the date of SARS-COV-2 introduction.

8. Sensitivity - Duration of immunity

To assess the sensitivity of our duration of immunity on the age-susceptibility to SARS-CoV-2, we reran the 2020 simulations, varying the duration of immunity parameter between 365 days and 3285 days. All other parameters were kept constant and the same samples were used as in the original analysis. Figure S8 shows the results. In all simulations, complete cross-protection resulted in a lower age susceptibility for children, however if the immunity was less than 2 years, the pattern of immunity was different. The simulations were not able to account for the reduced susceptibility in children in any scenario.

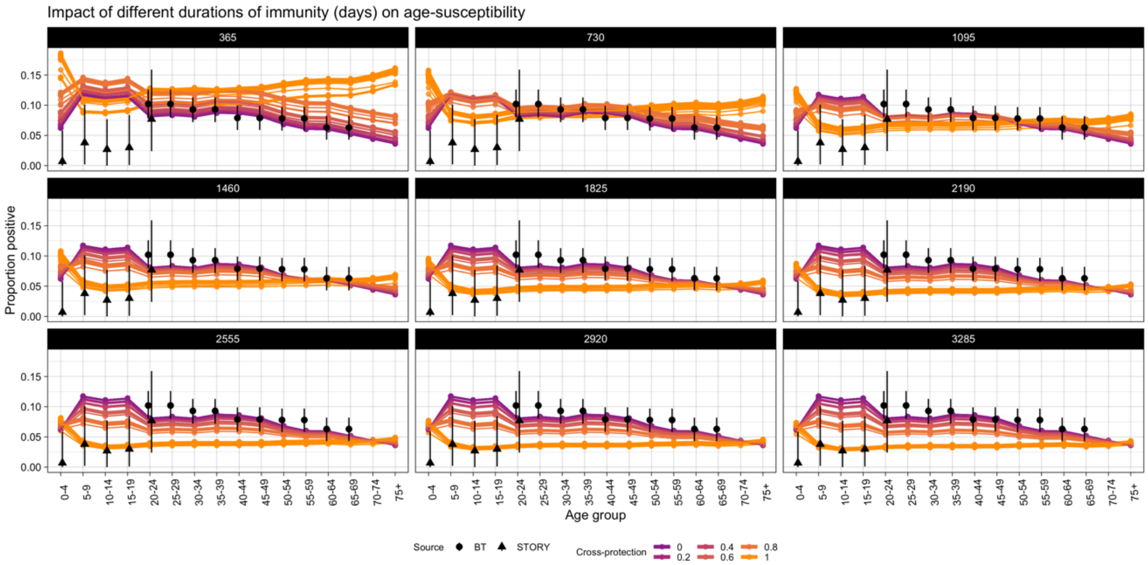

Figure S8: Age-specific serology. Simulated age-specific serology rates for SARS-CoV-2 by the end of May 2020. Each facet was run with a different duration of protection, displayed in the facet title, in days.

9. Sensitivity - Excluding 2014 season

As the 2014/15 season has a lower epidemic peak than the other seasons, we ran a sensitivity analysis by excluding data before August 2015. This is to take into account potential reduced testing in the first season. As previously, we ran the parallel tempering algorithm and removed 50,000 samples as burn in, resulting in 50,000 samples. The posterior parameter estimates differed slightly compared to the main model (Table S4), although both the estimate of the R0 and the waning parameter were still substantially higher than previous estimates. As before, we took samples from the posterior (in this case 50), and modelled the impact on age-susceptibility to SARS-CoV-2, with varying degrees of cross-protection. The conclusions remained the same as in the main paper, where cross-protection was unable to explain the reduced susceptibility of children.

Table S4: Posterior parameter estimates for the sensitivity analysis excluding all data before August 2015.

Parameter	Symbol	Median (95% CI) - excluding 2014/15	**Median (95% CI) - main model**
Basic Reproduction number	$R_{0,HCoV}$	3.7 (3.6 - 3.8)	5.9 (5.5-6.2)
Immunity duration	ω	4.4 (4.3 - 4.6)	7.8 (6.3 - 8.2)
Age-specific reporting proportion 0-4	$\mu_{HCoV,i}$	0.00097 (0.00094 - 0.0010)	0.00096 (0.00087 - 0.00100)
Age-specific reporting proportion 5 - 14, 45-64	$\mu_{HCoV,i}$	0.00015 (0.00014 - 0.00015)	0.00019 (0.00016 - 0.00020)

Age-specific reporting proportion 15-44	$\mu_{HCoV,i}$	0.000097 (0.000092 - 0.00010)	0.00013 (0.00011 - 0.00014)
Age-specific reporting proportion 65+	$\mu_{HCoV,i}$	0.00037 (0.00036 - 0.00038)	0.00058 (0.00043 - 0.00061)
Seasonal forcing amplitude	A	0.42 (0.040 - 0.43)	0.58 (0.42 - 0.61)
Seasonal forcing timing	ϕ	-39 (-39 - -38)	-35.1 (-36.2 - -24.1)

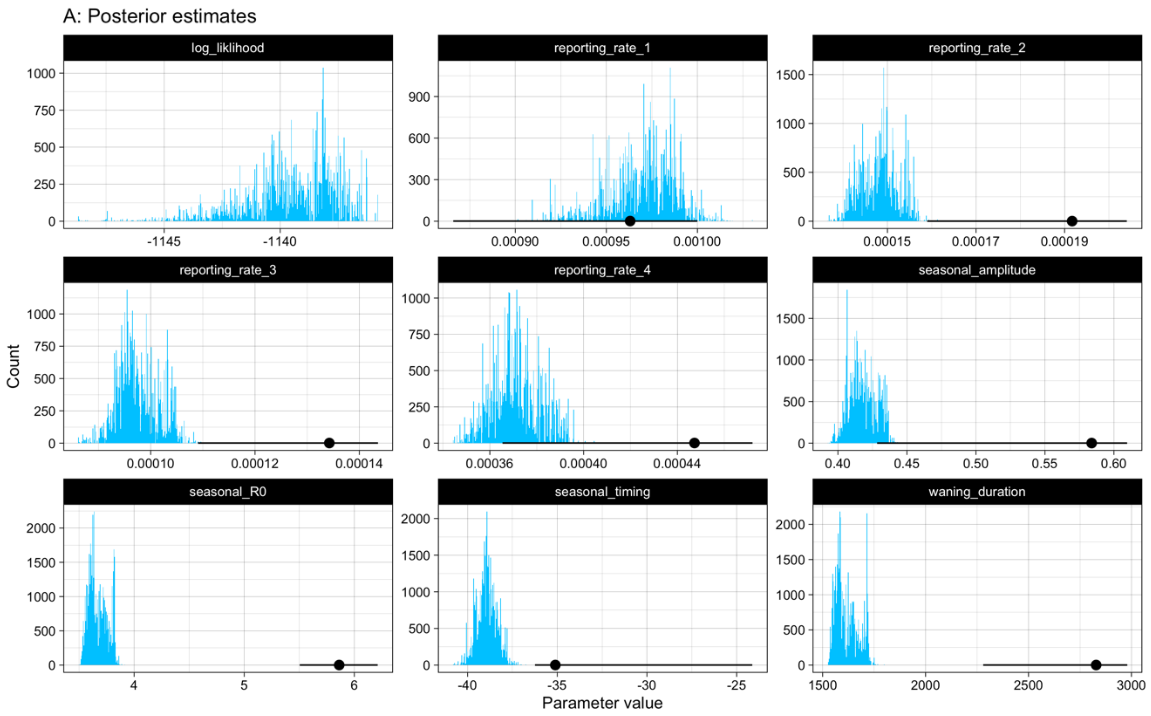

Figure S9: Posteriors from sensitivity analysis excluding 2014/2015 season. A) Histogram of posterior estimates from the beta-coronavirus only sensitivity analysis. Black lines show the median and 95%CI from the main model.

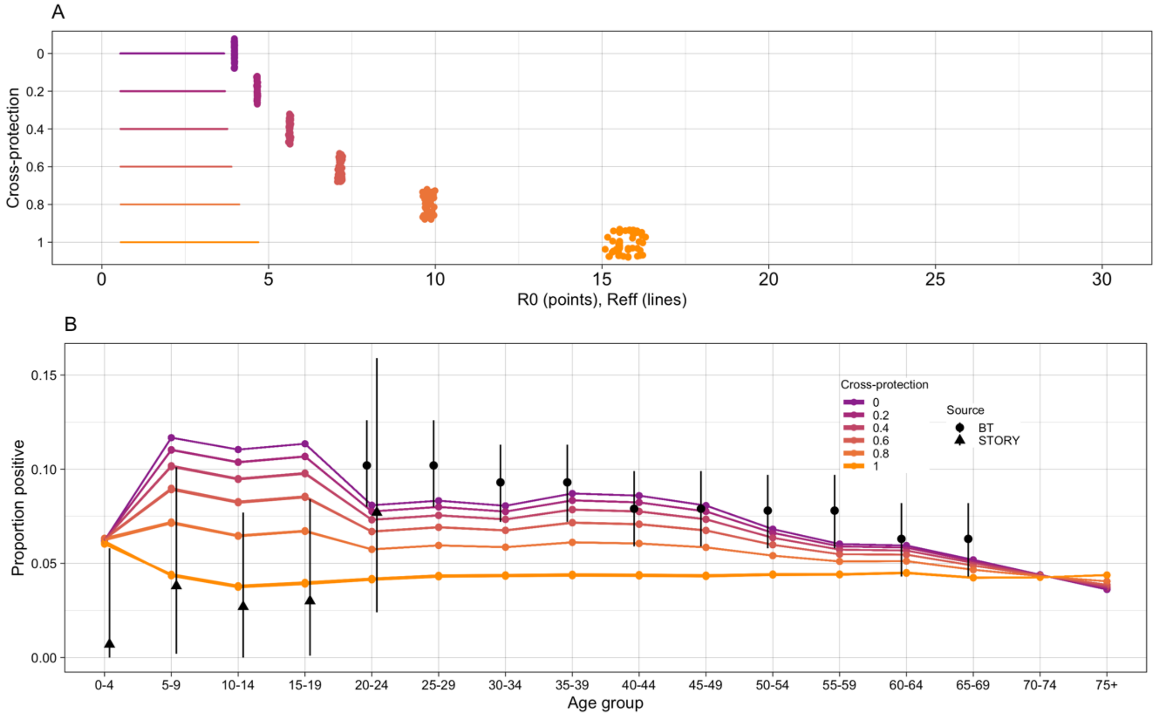

Figure S10: Simulations excluding 2014/15 season. A) Estimated R_0 values for SARS-CoV-2 with different strengths of cross-protection. Points display the $R_{0,\,C19}$ and lines show the range of $R_{eff,\,C19}$ during the simulation. B) Simulated age-specific serology rates for SARS-CoV-2 by the end of May 2020.

10. Sensitivity - Only beta-coronaviruses

Due to the complexities of cross-subtype immunity, we ran a sensitivity analysis including only beta-coronaviruses. We fixed the number of beta-coronaviruses at 54% of the overall number of coronaviruses each month, based on subtype-specific surveillance data from 2005-2017 in Glasgow[14]. Case numbers were rounded to the nearest full number. We reran the model fit with parallel tempering (50’000 burn-in, 50’000 samples), and the estimated posterior parameters are shown in Figure S10A. The key parameters of the seasonal HCoV R_0 and the duration of immunity overlapped with estimates from the main model (Figure S12). However in this sensitivity analysis parameters, especially the R_0 , appear bimodal. Figure S10B shows that these two modes have equivalent likelihood, with one mode coinciding with the estimated posterior R_0 from the main analysis. We ran the SARS-CoV-2 simulations with 50 samples from these new posteriors and found the same message: cross-protection was unable to explain the reduced susceptibility of children. (Figure S11).

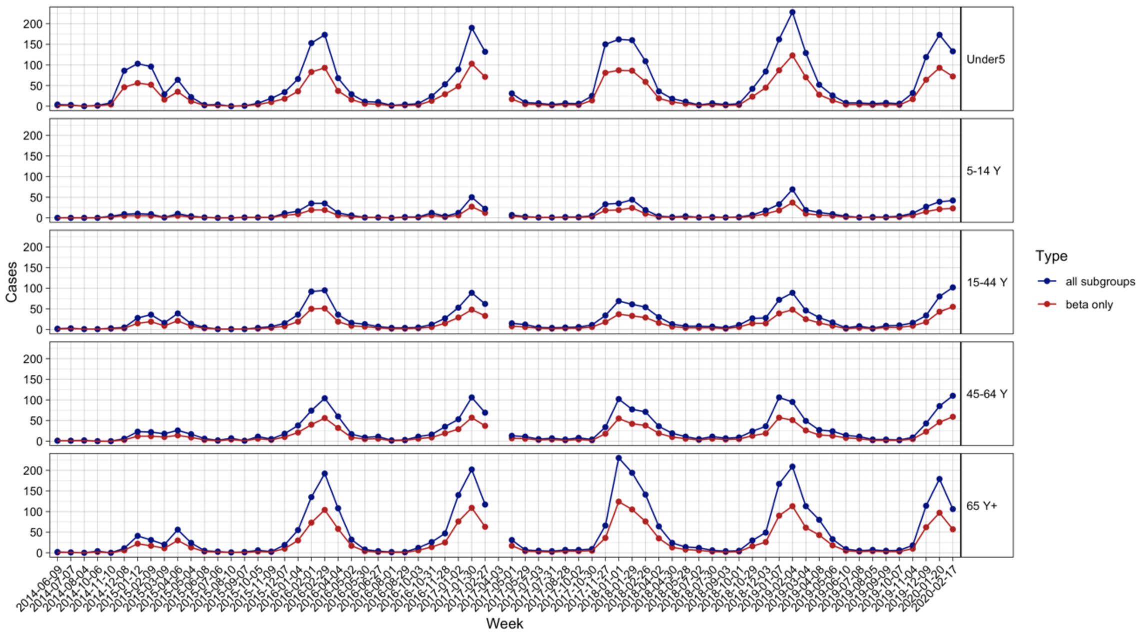

Figure S11: Beta-coronavirus only data. Blue shows the original data including all subtypes, red shows the data following our assumption that only 54% of cases are beta-coronavirus cases.

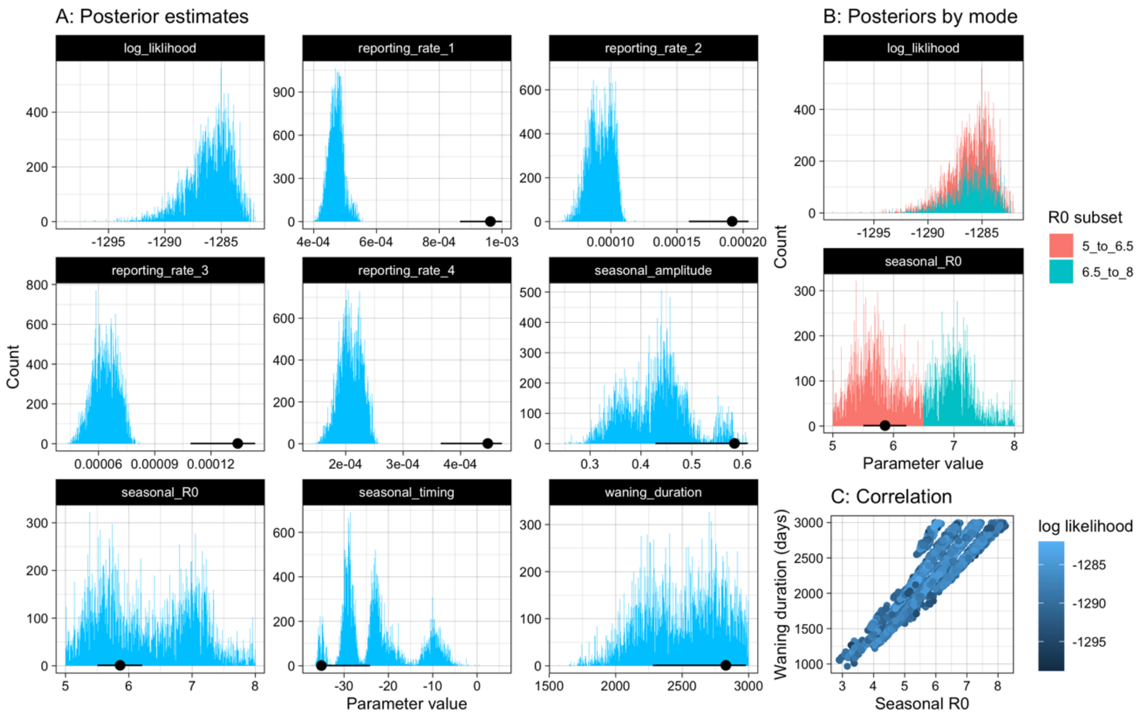

Figure S12: Posteriors from beta-coronavirus sensitivity analysis. A) Histogram of posterior estimates from the beta-coronavirus only sensitivity analysis. Black lines show the median and 95%CI from the main model. B) Subset of parameters showing histograms of the posterior distribution split by R_0 mode. Black line shows the median and 95%CI from the main model. C) Correlation between posterior samples of Seasonal HCoV R_0 and the waning duration.

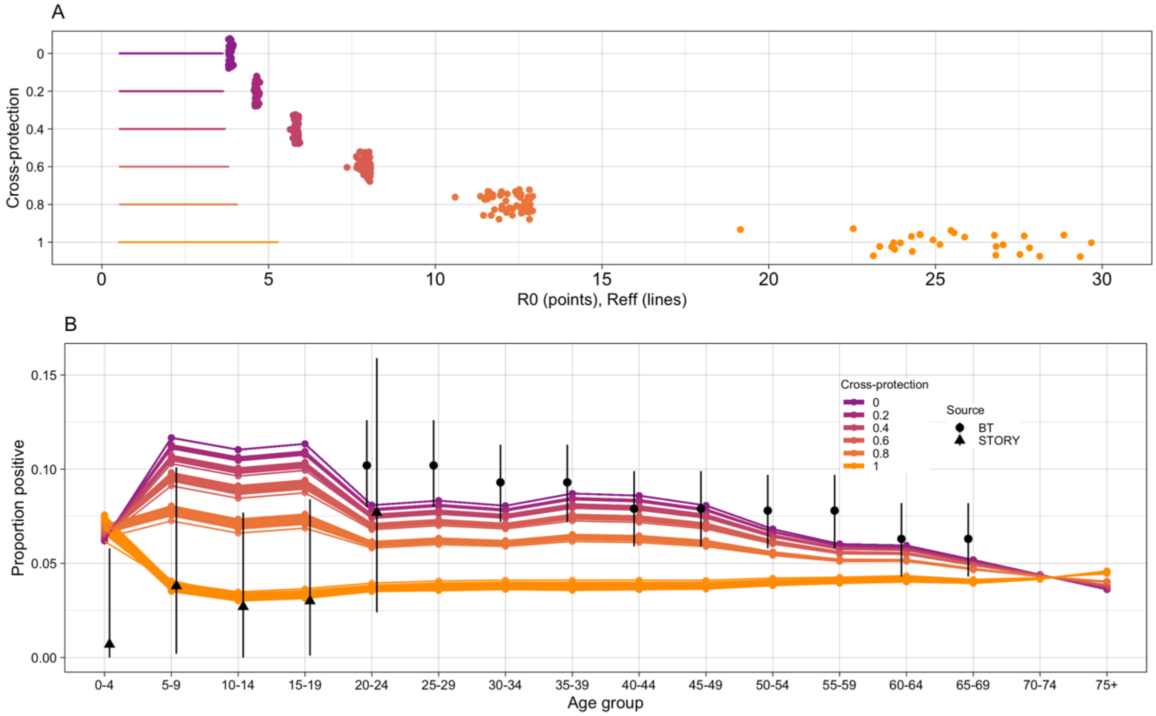

Figure S13: Simulations excluding alpha-coronaviruses. A) Estimated R_0 values for SARS-CoV-2 with different strengths of cross-protection. Points display the $R_{0,\,C19}$ and lines show the range of $R_{eff,\,C19}$ during the simulation. B) Simulated age-specific serology rates for SARS-CoV-2 by the end of May 2020.

11. Comparison with existing estimates

As our estimate of the duration of immunity is longer than other estimates, we compared it to parameters estimated in the 2020 Kissler et al. paper[1]. While the states are the same in the two model, there are some key differences including:

- Kissler et al. only model beta-coronaviruses, whereas we model all seasonal coronaviruses
- Kissler et al. model the two coronaviruses separately and estimate the cross-protection between them. We are instead modelling all coronaviruses together, thereby implicitly assuming complete cross-protection.
- The Kissler et al. model is not age structured
- The latency period in the Kissler et al. model is slightly longer (3 days instead of 2 days).

We investigated the impact of using their estimated parameter values in our model. We fixed their estimated values of R_0 (2), waning (45 weeks) and seasonality parameters, as well as changing our model to match their longer latency period. We then fit the reporting rates using Maximum Likelihood Estimation (L-BFGS-B optimisation) to fit the seasonal coronavirus, using the same likelihood as in the main paper. While the model still produced what looked like a good fit (See Fig S4), the log likelihood values were significantly lower (-2235 vs -1905).

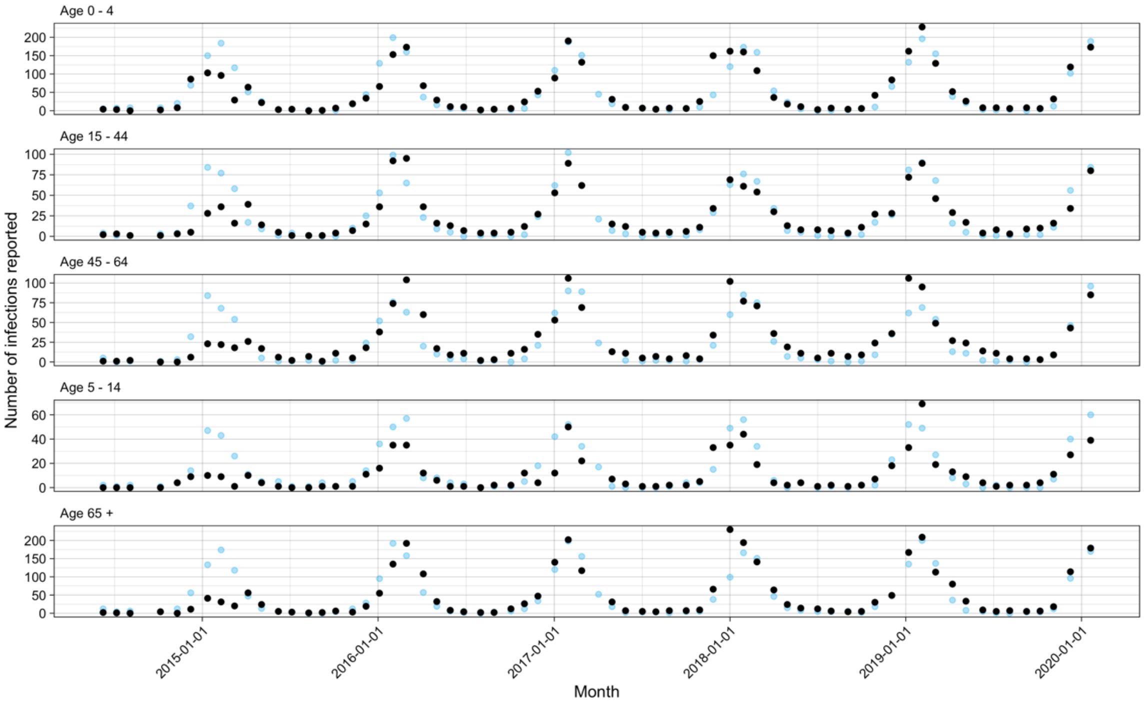

Figure S14: Model fit for seasonal HCoV, using the immunity and R_0 parameters from Kissler et al. 2020.

References

1. Kissler, S. M. Projecting the transmission dynamics of SARS-CoV-2 through the postpandemic period | Science. https://science.sciencemag.org/content/368/6493/860.
2. Liu, Z., Chu, R., Gong, L., Su, B. & Wu, J. The assessment of transmission efficiency and latent infection period in asymptomatic carriers of SARS-CoV-2 infection. *Int. J. Infect. Dis. IJID Off. Publ. Int. Soc. Infect. Dis.* **99**, 325–327 (2020).
3. Tyrrell, D. A., Cohen, S. & Schlarb, J. E. Signs and symptoms in common colds. *Epidemiol. Infect.* **111**, 143–156 (1993).
4. Birrell, P. J. *et al.* Forecasting the 2017/2018 seasonal influenza epidemic in England using multiple dynamic transmission models: a case study. *BMC Public Health* **20**, (2020).
5. Davies, N. G. *et al.* Estimated transmissibility and impact of SARS-CoV-2 lineage B.1.1.7 in England. *Science* (2021) doi:10.1126/science.abg3055.
6. Byrne, A. W. *et al.* Inferred duration of infectious period of SARS-CoV-2: rapid scoping review and analysis of available evidence for asymptomatic and symptomatic COVID-19 cases. *BMJ Open* **10**, e039856 (2020).
7. Levin, A. T., Cochran, K. B. & Walsh, S. P. Assessing the Age Specificity of Infection Fatality Rates for COVID-19: Meta-Analysis & Public Policy Implications. *medRxiv* 2020.07.23.20160895-2020.07.23.20160895 (2020) doi:10.1101/2020.07.23.20160895.
8. ONS. Births in England and Wales: 2019.
9. Estimates of the population for the UK, England and Wales, Scotland and Northern Ireland - Office for National Statistics. https://www.ons.gov.uk/peoplepopulationandcommunity/populationandmigration/populationestimates/datasets/populationestimatesforukenglandandwalesscotlandandnorthernireland.
10. Mossong, J. *et al.* Social contacts and mixing patterns relevant to the spread of infectious diseases. *PLoS Med.* **5**, 0381–0391 (2008).
11. Diekmann, O., Heesterbeek, J. A. P. & Roberts, M. G. The construction of next-generation matrices for compartmental epidemic models. *J. R. Soc. Interface* **7**, 873–85 (2010).
12. Vousden, W. D., Farr, W. M. & Mandel, I. Dynamic temperature selection for parallel tempering in Markov chain Monte Carlo simulations. *Mon. Not. R. Astron. Soc.* **455**, 1919–1937 (2016).
13. Vats, D. & Knudson, C. Revisiting the Gelman-Rubin Diagnostic. (2018).
14. Nickbakhsh, S. *et al.* Epidemiology of Seasonal Coronaviruses: Establishing the Context for the Emergence of Coronavirus Disease 2019. *J. Infect. Dis.* **222**, 17–25 (2020).

CMMID Working Group Authors

The following authors were part of the Centre for Mathematical Modelling of Infectious Disease COVID-19 Working Group. Each contributed in processing, cleaning and interpretation of data, interpreted findings, contributed to the manuscript, and approved the work for publication:

Rachael Pung, Paul Mee, William Waites, Damien C Tully, Katherine E. Atkins, C Julian Villabona-Arenas, Graham Medley, Frank G Sandmann, Anna M Foss, Sophie R Meakin, Carl A B Pearson, Emilie Finch, Nikos I Bosse, Christopher I Jarvis, Kiesha Prem, Alicia Rosello, Kevin van Zandvoort, Rosanna C Barnard, Jiayao Lei, Yang Liu, Adam J Kucharski, Ciara V McCarthy, Sam Abbott, Emily S Nightingale, Joel Hellewell, Thibaut Jombart, David Hodgson, Gwenan M Knight, Amy Gimma, Yung-Wai Desmond Chan, Yalda Jafari, Samuel Clifford, Timothy W Russell, Fiona Yueqian Sun, Simon R Procter, Akira Endo, Oliver Brady, Kaja Abbas, Billy J Quilty, Mark Jit, Sebastian Funk, Fabienne Krauer, Matthew Quaife, Hamish P Gibbs, W John Edmunds, Mihaly Koltai, Kathleen O'Reilly, Rachel Lowe, James D Munday.
